# Molecular typing and antimicrobial susceptibility profiles of *Campylobacter jejuni* and *Campylobacter coli* Isolates from Patients and raw meat in Huzhou, China, 2021–2022

**DOI:** 10.1371/journal.pone.0311769

**Published:** 2024-12-11

**Authors:** Xiaofang Wu, Chen Liping, Fenfen Dong, Wei Yan, Yuehua Shen, Lei Ji

**Affiliations:** Huzhou Center for Disease Control and Prevention, Huzhou, China; Tribhuvan University, NEPAL

## Abstract

**Background:**

*Campylobacter* species are zoonotic pathogens, and are considered to be the major foodborne pathogen that causes outbreaks and sporadic gastrointestinal illnesses both in developed and developing countries. In this study, the molecular typing and antimicrobial susceptibility profiles of *Campylobacter jejuni* and *Campylobacter coli* isolates from patients and raw meat between 2021 and 2022 in Huzhou were analyzed by using pulsed-field gel electrophoresis (PFGE), multilocus sequence typing (MLST) and antimicrobial susceptibility testing.

**Methods:**

From September 1, 2021 to December 31, 2022, a total of 342 fecal specimens from diarrheal patients at a sentinel hospital in Huzhou and 168 samples of raw meat products collected from farmers’ markets and supermarkets, were subjected to *Campylobacter* isolation and identification. The agar dilution method was used to determine resistance of the *Campylobacter* isolates to eleven antibiotics. In addition, pulsed-field gel electrophoresis (PFGE) and multilocus sequence typing (MLST) were performed to compare their genetic relationships.

**Results:**

78 *Campylobacter* isolates were recovered, comprising 58 isolates (74.36%, 58/78) of *Campylobacter jejuni* (34 patient isolates and 16 food isolates) and 20 isolates (25.64%, 20/78) of *Campylobacter coli* (6 patient isolates and 14 food isolates). *Campylobacter* has emerged as a predominant foodborne pathogen in the local region, with detection rate reached 11.70% among 342 diarrhea samples. The *Campylobacter* isolation rate in 168 raw meat was 22.62% (38/168), all originating from poultry meat, with chicken been the major source of infection (86.84%, 33/38). Both PGFE type and MLST data confirmed that *Campylobacter* stains circulating in Huzhou are genetically diverse, with *Campylobacter jejuni* isolates being more diverse than *Campylobacter Coli*. PFGE typing revealed 45 band patterns among 54 *Campylobacter jejuni* strains and 17 band patterns among 19 *Campylobacter Coli* strains. 50 *Campylobacter jejuni* strains from different sources were classified into 37 ST types, showing a dispersed distribution and encompassing over 12 clonal complexes (CCs), with CC-21 being the most prevalent CC (22.00%, 11/50). The distribution of ST types in the 18 *Campylobacter Coli* strains was relatively concentrated, with 83.33% (15/18) of isolates belonging to the CC-828. In this study, 2 groups of *Campylobacter jejuni* strains (PFGE J2-ST464 and PFGE J9-ST-2328) originated from humans and chickens showed high genetic homologies by comparing PFGE and MLST results. Besides, some disagreement between PFGE and MLST was observed for certain ST, indicating a weak correlation between PFGE and MLST for certain *Campylobacter* strains. Most of the *Campylobacter* isolates were highly resistant to nalidixic-acid, ciprofloxacin and tetracycline. The multiple antibiotic resistance of *Campylobacter Coli* (89.47%) is higher than *Campylobacter jejuni* (29.63%).

**Conclusion:**

*Campylobacter* is an important foodborne pathogen in both diarrheal patients and raw meat products in Huzhou City, exhibiting multiple antibiotic resistance and high level of genetic diversity.

## Introduction

*Campylobacter*, a zoonotic pathogen, can cause symptoms such as diarrhea, fever, and abdominal pain. Additionally, it can lead to severe complications like Guillain-Barré syndrome and reactive arthritis, posing a significant threat to human health [1]. In developed countries, Campylobacter infection has become more prevalent than infections caused by pathogens such as Salmonella, E. coli and, Vibrio parahaemolyticus, establishing it as the leading cause of bacterial diarrhea worldwide [2]. *Campylobacter jejuni (C*. *jejuni)* and *Campylobacter coli (C*. *coli)* are the main *Campylobacter* species that cause gastroenteritis in humans and responsible for approximately 95% of all Campylobacter infections in developing countries [3]. It has been reported that globally, there are 400 to 500 million cases of diarrhea annually caused by *C*. *jejuni*, making it a serious public health concern [4]. *Campylobacter* widely inhabits the human and animal intestines [5]. Poultry is a significant source of contamination in the human food chain. During poultry farming, poultry infected with *Campylobacter* do not exhibit any clinical symptoms but can continuously shed the bacteria into the environment and carry it for life [6]. This can easily lead to cross-contamination between poultry and livestock and its products during slaughter, processing, and retail stages [7]. Epidemiological studies have shown that up to 30% of human Campylobacter infections are caused by handling, preparing, and consuming raw or undercooked poultry. Poultry meat—especially chicken meat—is the most common source of infection in humans, along with other insufficiently heated meat, raw milk, and contaminated water [8–11].

With the increasing awareness of the major public health importance of *Campylobacter*, studies about the prevalence of *Campylobacter* isolated from clinical cases in China have been carried out in the recent years. However, only a few studies have investigated the isolation rate and molecular characterization of *Campylobacter* spp. from both food and human clinical sources in China. The link between foodborne and human clinical isolates of *Campylobacter* has remained largely uncharacterized. The aim of this study was to investigate the molecular typing and antimicrobial susceptibility profiles of *C*. *jejuni* and *C*. *coli* isolates from patients and raw meat in Huzhou and to evaluate the phylogenetic relationships of *Campylobacter* strains from human patients and raw meat products using PFGE and MLST methods.

## Materials and methods

### Ethics statement

The protocol was approved by the ethics committee of Huzhou Center for Disease Control and Prevention (approval number: HZ2021005). The only human material used in this study is fecal specimen from outpatient with acute diarrhea for local foodborne disease surveillance project, data records and collected clinical specimens were deidentified and anonymous. Patient consent was not required as the research results will not be used as a basis for any auxiliary diagnosis or for any commercial purposes. Furthermore, any identifiers related to participants will be removed from the research results to ensure that personal privacy is not compromised. Therefore, there is no objective risk to the participants.

### Sample collection and strain isolation

According to the guidelines of the local foodborne disease surveillance project in Huzhou, from September 1, 2021 to December 31, 2022, a total of 342 fecal specimens from outpatients with acute diarrhea at the sentinel hospital and 168 samples of raw meat products (50 samples of livestork meat products and 118 samples of raw poultry products) purchased from farmers’ markets and supermarkets, were subjected to *Campylobacter* isolation. 168 raw meat products mainly refer to fresh poultry, frozen poultry, and fresh livestork meat, including 25 portions of raw pork and 25 portions of raw beef, 30 ducks, and 88 chickens.

For *Campylobacter* spp. isolation, raw meat was first placed in sterile self-sealing bags containing 500 ml of BPW culture medium, followed by vigorous rubbing for 5 minutes. Then a *Campylobacter* isolation kit incorporating a membrane filter method (ZC-CAMPY-001 for specimens and ZC-CAMPY-002 for meat, Qingdao Sinova Biotechnology Co., Ltd., Qingdao, China) was used to isolate *Campylobacter*. Briefly, 2 mL of meat suspension and suitable amount of fecal specimen was transferred to 4 mL of growth-promoting enrichment Preston broth provided in the kit. The enrichment broth was then incubated at 42°C under microaerobic conditions (5% O2, 10% CO2, and 85% N2) for 24 hours. Three hundred microliters drop of the enrichment broth were applied to the 0.45-μm pore-size filter and left on the surface of Karmali and Columbia blood agar plates. After 30 minutes, the filters were removed, and these plates were further incubated at 42°C under microaerobic condition. Suspicious colonies were subcultured, and identified using matrix-assisted laser desorption/ionization time of flight (MALDI-TOF) mass spectrometry (VITEK MS).

### PFGE molecular typing

Pulsed-field gel electrophoresis (PFGE) molecular typing was performed according to the PulseNet standardized protocol for *C*. *jejuni* (Available online: https://www.cdc.gov/pulsenet/PDF/campylobacter-pfge-protocol-508c.pdf). In brief, genomic DNA was digested with SmaI (Takara, Dalian, China), and run on a CHEF Mapper PFGE system (Bio-Rad Laboratories, Hercules, CA) for 16 h on SeaKem gold agarose (Lonza, Rockland, MD, USA) in 0.5×Tris-borate-EDTA. XbaI-digested DNA from *Salmonella* enterica serovar Braenderup H9812 was used as the standard size. *Salmonella* enterica serovar Braenderup digested with XbaI (Takara) was used as the molecular reference marker. The gel images were stored electronically as TIFF files and bands were analyzed by using BioNumerics software v. 7.6 (Applied Maths, Kortrijk, Belgium). The similarity between chromosomal fingerprints was scored using the Dice coefficient.

### MLST molecular typing

Multilocus sequence typing (MLST) was performed by sequencing seven housekeeping loci (aspA, glnA, gltA, glyA, pgm, tkt, and uncA) according to previously described primers for *C*. *jejuni* and *C*. *coli* (https://pubmlst.org/organisms/*Campylobacter*-jejunicoli/primers). The nucleotide sequences of the amplicons were submitted to the pubMLST database (https://pubmlst.org/) for online data analysis, resulting in corresponding Sequence Types (ST) and Clonal Complexes (CC). For ST types not found in the database, new ST types were applied for based on strain sequences using blast. A minimum spanning tree (MST) and dendrogram of MLST data was created using BioNumerics v.7.6 (Applied Maths, Kortrijk, Belgium).

### Antibiotic susceptibility testing

Antibiotic susceptibility testing was conducted using the agar dilution method recommended by the Clinical and Laboratory Standards Institute (CLSI) using a commercial kit (ZC-AST-001, Zhongchuang Biotechnology Ltd. Corp., Qingdao, China). The antibiotics tested included macrolides: erythromycin (ERY) and azithromycin (AZI); quinolones and fluoroquinolones: nalidixic acid (NAL) and ciprofloxacin (CIP); aminoglycosides: gentamicin (GEN) and streptomycin (STR); chloramphenicol: chloramphenicol (CHL) and florfenicol (FLO); tetracyclines: tetracycline (TET); ketolides: telithromycin (TEL); lincosamides: clindamycin (CLI). MICs were interpreted in accordance with the standard of National Antimicrobial Resistance Monitoring System (NARMS-2014). The breakpoints for resistance were as follows: ERY ≥ 32 μg/mL, AZI ≥ 1 μg/mL, NAL ≥ 32 μg/mL, CIP ≥ 4 μg/mL, GEN ≥ 4 μg/mL, STR ≥ 16 μg/mL, CHL ≥ 32 μg/mL, FLO ≥ 8 μg/mL, TET ≥ 16 μg/mL, TEL ≥ 8 μg/mL, CLI ≥ 1 μg/mL. Quality control was performed with *C*. *jejuni* ATCC 33560. The multiple antibiotic resistance index (MARI) was used to quantify the multi-resistance of *Campylobacter* isolates. MAR index = a/b. In this formula, “a” indicated the number of antibiotics to which the isolate was resistant and “b” indicated the total number of antibiotics to which the isolate was tested. Multi-drug resistance (MDR) was defined as resistance to three or more classes of antimicrobials in this study.

### Statistical analysis

Statistical analysis was performed using SPSS 19.0 software. The χ2 test was employed, and significance was determined at P < 0.05.

## Results

### Prevalence of *Campylobacter* spp. in Huzhou

We collected 342 fecal specimens from diarrheal patients and 168 samples of raw meat products from farmers’ markets and supermarkets during 2021 and 2022. Seventy eight *Campylobacter* isolates were recovered, comprising 58 isolates of *C*. *jejuni* (74.36%, 58/78) and 20 isolates of *C*.*coli* (25.64%, 20/78). The isolation rate of *Campylobacter* in diarrhea patients was 11.70% (40/342), with detection rates of *C*. *jejuni* and *C*.*coli* at 9.94% (34/342) and 1.75% (6/342), respectively. The isolation rate of *Campylobacter* in raw meat products was 22.62% (38/168), with all strains isolated from poultry meat (5 from duck and 33 from chicken). A significant higher percentage (P = 0.035,χ2 = 4.4476) of *Campylobacter* spp. isolates was observed in chicken samples (37.50%, 33/88) as compared to those in duck samples (16.67%, 5/30). The detection rate for *C*. *jejuni* in raw meat was 14.29% (24/168), while *C*.*coli* had a detection rate of 1.75% (6/342). The comparison of detection rates between *C*. *jejuni* and *C*.*coli* in samples from diarrhea patients showed statistically significant differences (χ2 = 20.81, P < 0.0001), see Table 1.

**Table 1:** Detection rate of *Campylobacter* in raw meat and diarrheal samples in Huzhou.

Sample Name	Number of Samples	*C*. *jejuni (*n/%)	*C*. *coli (*n/%)	Total	χ2	P
Diarrheal specimens	342	34/9.94	6/1.75	40/11.70	20.8174	<0.0001
Raw meat	168	24/14.29	14/8.33	38/22.62	2.9671	0.085
Total	510	58/11.37	20/3.92	78/15.29		

### PFGE clustering

**Fig 1:**
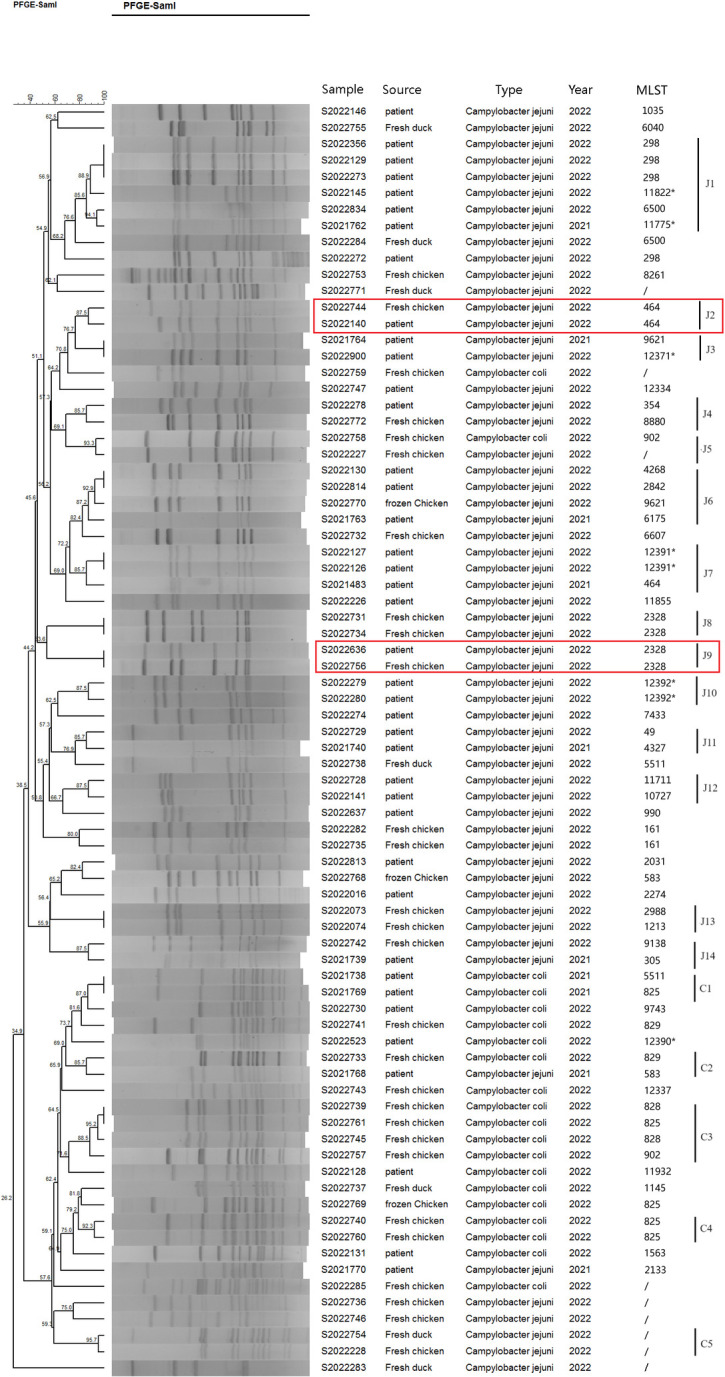
Dendrogram of 73 *Campylobacter* strains based on SmaI-mediated PFGE profiles. Sample ID, strain source, isolation year, MLST type are depicted on the right. Asterisk indicates newly designated ST in this study. Fifteen groups of closely related C. jejuni isolates (band pattern similarity greater than 85.0%) were assigned as J1-J15, while five groups of closely related C. coli isolates (band pattern similarity greater than 85.0%) were assigned as C1-C5. Two groups (PFGE J2-ST464 and PFGE J9-ST-2328) of *C*. *jejuni* strains originated from humans and chickens which were confirmed to be clonally related by comparing PFGE and MLST results are marked with red rectangle.

A total of 78 *Campylobacter* strains were subjected to PFGE after digestion with the restriction enzyme Sma I and 73 valid profiles were obtained, comprising 54 *C*. *jejuni* strains (34 from patients and 20 from food samples) and 19 *C*.*coli* strains (6 from patients and 13 from food samples). Cluster analysis revealing a band pattern similarity of 26.8% to 100% among the 73 *Campylobacter* isolates, see Fig 1.

Among the 54 *C*. *jejuni* strains, a total of 45 band patterns were obtained, with 15 groups of closely related isolates (greater than 85.0% similarity in banding patterns) which were assigned a profile group number (J1–J15). The most common profile group was J1, which included 6 isolates from diarrhoea patient. 4 groups had both patient and food isolates (J2, J4, J6, J9) and only 1 group (J9) each of patient isolate (ID 2022636) and food isolate (ID 2022756) had identical profiles. 17 band patterns were obtained from the 19 *C*.*coli* isolates by SmaI digestion analysis. 5 groups were identified that had isolates possessing over 85% similarities with each other and were designated C1–C5. The most common profile group was C3, which included 6 food isolates. Only one group (C2) comprised both patient and food isolates and shared 85.7% similarities with each other. No isolates from patients formed identical PFGE patterns with chicken or duck among *C*. *coli* strains.

### MLST typing

50 *C*. *jejuni* strains (34 patient isolates and 16 food isolates) and 18 *C*. *coli* strains (6 patient isolates and 12 food isolates) were selected for MLST molecular typing, with the results presented in Table 2. We identified 37 STs of 12 CCs among the 50 *C*. *jejuni* isolates, including 5 STs (ST-11775, ST-11822, ST-12371, ST-12391, ST-12392) were newly designated in this study. 13 STs from 17 isolates did not assign to any known CCs. The most commonly isolated CC was CC-21 (22.00%, 11/50), followed by CC-353(6.00%, 3/50), CC-464(6.00%, 3/50) and CC-574 (6.00%, 3/50). The most common STs in patients and food were ST-298(4 strains) and ST-2328 (3 strains), respectively. STs overlapped in both patients and food isolates included ST-6500, ST-464, ST-9621 and ST-2328. MLST analysis for the 18 *C*.*coli* isolates resulted in 11 STs, of which 1 ST (ST12390) was new. Except for 3 unclassified STs (ST-1145, ST-11932 and ST-12337), all other STs were classified into the same clonal complex, CC-828. STs overlapped in both patients and food isolates for *C*.*coli* in this study was ST-825. MLST data summary of 50 *C*. *jejuni* strains and 18 *C*. *coli* strains in this study were presented in S1 Table.

**Table 2:** MLST typing results of *Campylobacter* isolates from patient and raw meat in Huzhou.

*C*. *jejuni (n* = 50)						*C*. *coli (n* = 18)		
CC	ST (n)	Source	CC	ST (n)	Source	CC type	ST (n)	Source
CC-21	298 (4)	Patient	CC-574	2031 (1)	Patient	CC-828	825 (1)	Patient
	6175 (1)	Patient		305 (1)	Patient		825 (4)	Poutry meat
	6500 (1)	Patient		8880 (1)	Poutry meat		828 (2)	Poutry meat
	6500 (1)	Poutry meat	CC-607	9621 (1)	Patient		829 (2)	Poutry meat
	8261 (1)	Poutry meat		9621 (1)	Poutry meat		902 (2)	Poutry meat
	11822* (1)	Patient	CC-1034	6040 (1)	Poutry meat		1563 (1)	Patient
	12392* (2)	Patient	unclassified	1035 (1)	Patient		5511 (1)	Patient
CC-45	12371* (1)	Patient		2133 (1)	Patient		9743 (1)	Patient
	583 (1)	Patient		2274 (1)	Patient		12390* (1)	Patient
CC-49	49 (1)	Patient		2328 (1)	Patient	unclassified	1145 (1)	Poutry meat
	11775* (1)	Patient		2328 (3)	Poutry meat		11932 (1)	Patient
CC-52	161 (2)	Poutry meat		4268 (1)	Patient		12337 (1)	Poutry meat
CC-257	990 (1)	Patient		4327 (1)	Patient			
CC-353	10727 (1)	Patient		6606 (1)	Poutry meat			
	7433 (1)	Patient		6607 (1)	Poutry meat			
	2842 (1)	Patient		9138 (1)	Poutry meat			
CC-354	354 (1)	Patient		11711 (1)	Patient			
	2988 (1)	Poutry meat		11855 (1)	Patient			
CC-460	1213 (1)	Poutry meat		12334 (1)	Patient			
CC-464	464 (2)	Patient		12391* (2)	Patient			
	464 (1)	Poutry meat						

Note

* denotes newly discovered ST types; the numbers in parentheses represent the quantity of *Campylobacter* strains.

**Fig 2:**
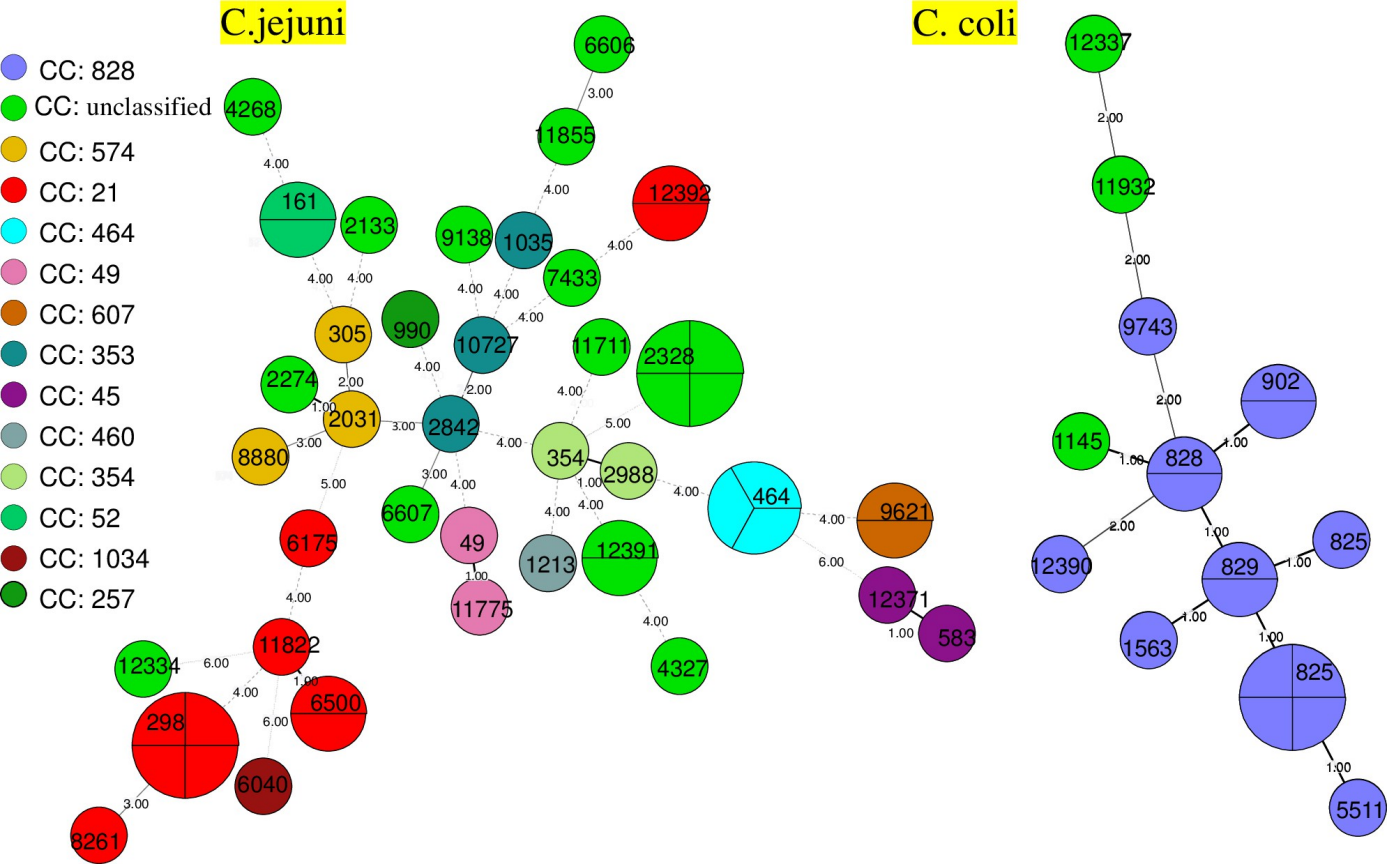
Minimum spanning tree of 50 *C*. *jejuni* strains and 18 *C*. *coli* strains. The isolates are featured by different color according to the corresponding CCs. Different circles correspond to different STs. Size of circle indicates number of isolates within the same ST, branches and numbers represent allelic differences between isolates.

**Fig 3:**
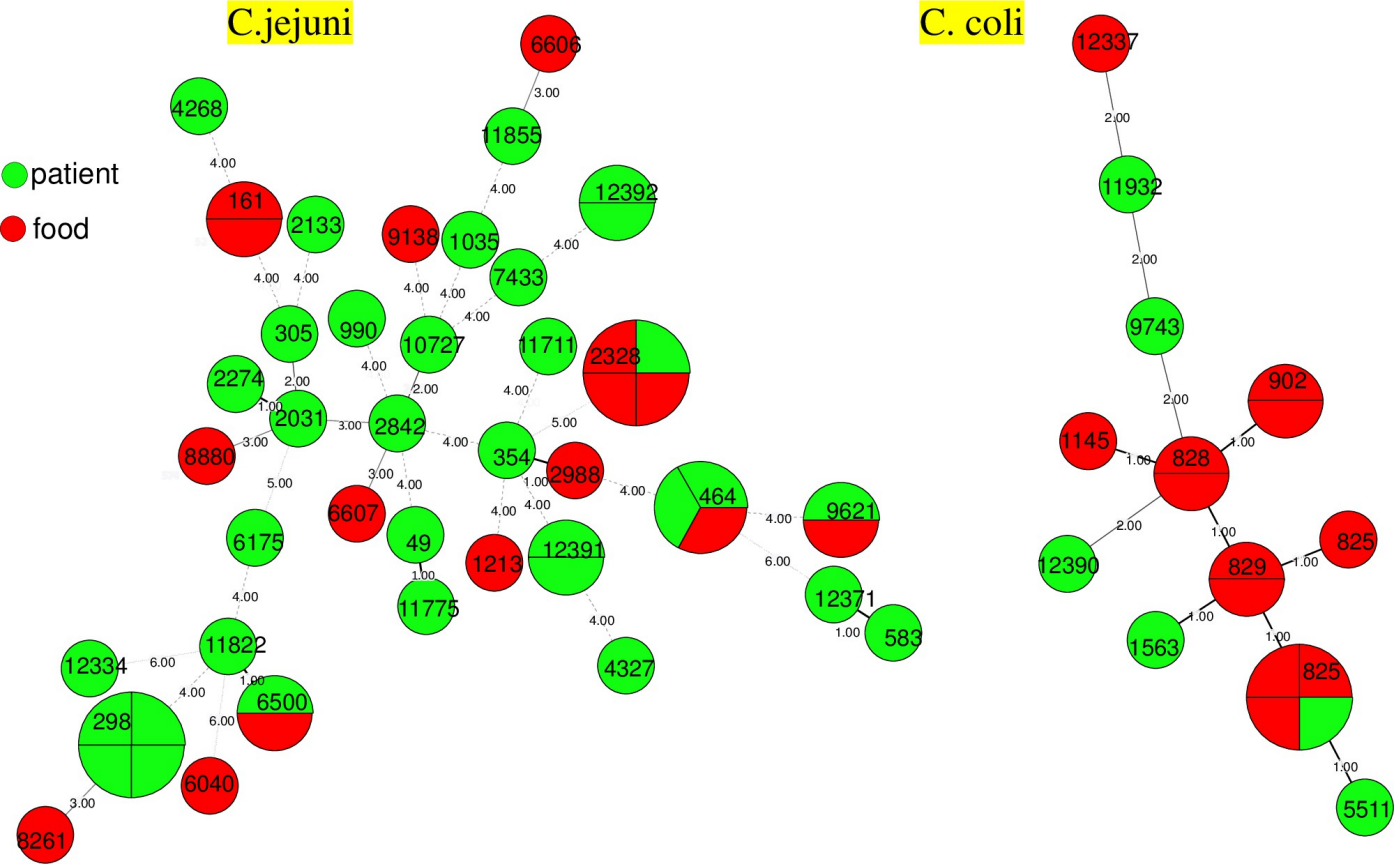
Minimum spanning tree of 50 *C*. *jejuni* strains and 18 *C*. *coli* strains. The isolates are featured by different color according to isolated sources. Different circles correspond to different STs. Size of circle indicates number of isolates within the same ST, branches and numbers represent allelic differences between isolates.

The minimum spanning trees based on MLST data for the cluster analysis of *C*. *jejuni* and *C*.*coli* isolates are depicted in Figs 2 and 3. The results revealed close genetic relationships among strains within the same CCs, see Fig 2. For instance, ST-11822, ST-298, and ST-6500 from the CC-21 were positioned on the same small branch. Similarly, within the CC-828, ST-829, ST-825, ST-1563 and ST-5511 displayed close genetic relationships. Strains from different CCs exhibited relatively distant genetic relationships. *C*. *jejuni* and *C*.*coli* from diarrheal patients and raw meat products demonstrated a dispersed distribution, with no significant clustering observed, See Fig 3.

Comparison of genetic relatedness between PFGE and MLST revealed that not all clustered PFGE types with 100.0% similarity in banding pattern showed identical ST types, while not all the isolates with the same STs shared 100.0% similarity in PFGE profile type, see Figs 1 and 2 groups (PFGE J2-ST464 and PFGE J9-ST-2328) of *C*. *jejuni* strains originated from humans and chickens were confirmed to be clonally related by comparing PFGE and MLST results.

### Antibacterial susceptibility of *C*. *jejuni* and *C*. *coli* isolates

Antibiotic susceptibility testing was conducted on 73 *Campylobacter* isolates, including 54 *C*. *jejuni* (34 from patient isolates and 20 from food isolates) and 19 *C*.*coli* strains (6 from patient isolates and 13 from food isolates), see Table 3. *C*. *jejuni* showed resistance most frequently to NAL (94.44%), followed by TET (88.89%), CIP (87.04%), CLI (16.67%), ERY /GEN/FLO (12.96%), AZM/TEL (11.11%), STR (9.26) and CHL (5.56%), while *C*. *coli* displayed highest resistance rate to NAL/CIP (94.74%), followed by TET (84.21%), ERY (63.16%), AZM (52.63%), TEL/CLI (42.11%), GEN (31.58%), STR (26.32), FLO (10.53) and CHL (5.26%). Compared with *C*. *jejuni*, statistical higher resistant rates were observed in *C*. *coli* for ERY (χ^2^ = 18.39, p < 0.0001), AMZ (χ^2^ = 14.16, p = 0.0001) and TEL (χ^2^ = 8.71, p = 0.003). Specifically, ERY resistance in *C*. *coli* (63.16%) is much more prevalent than that in *C*. *jejuni* (12.96%). The MARI of the tested *C*. *jejuni and C*. *coli* isolates from the current study are presented in Tables 4 and 5, respectively. A total of 18 different antibiotic resistance patterns with MARI ranging from 0.18 to 0.91 were observed in 54 *C*. *jejuni* isolates, while a total of 12 different antibiotic resistance patterns with MARI ranging from 0.09 to 0.73 were observed in 19 *C*. *coli* isolates. The MDR rate were 29.63% (16/54) for *C*. *jejuni* and 89.47% (17/19) for *C*. *coli*. The difference in MDR rates between the two groups was statistically significant (χ^2^ = 20.321, P < 0.01).

**Table 3:** Antimicrobial resistant rate of 54 *C*. *jejuni* and 19 *C*. *coli* isolates from different sources against 11 antimicrobials.

Classes	Antibiotic	*C*. *jejuni*			*C*. *coli*		
		Patient strains(n = 34)	Food strains(n = 20)	Totle(n = 54)	Patient strains(n = 6)	Food strains(n = 13)	Totle(n = 19)
		N/Resistance Rate (%)	N/Resistance Rate (%)	N/Resistance Rate (%)	N/Resistance Rate (%)	N/Resistance Rate (%)	N/Resistance Rate (%)
Macrolides	ERY	6/ 17.65	1/5.00	7/12.96	5/83.33	7/53.85	12/63.16
	AZM	4/ 11.76	2/10.00	6/11.11	3/50.0	7/53.85	10/52.63
Quinolones	NAL	31/91.18	20/100.00	51/94.44	5/83.33	13/100.00	18/94.74
	CIP	28/82.35	19/95.00	47/87.04	5/83.33	13/100.00	18/94.74
Aminoglycosides	GEN	4/11.76	3/15.00	7/12.96	1/16.67	5/38.46	6/31.58
	STR	4/11.76	1/5.00	5/9.26	1/16.67	4/30.77	5/26.32
Chloramphenicol	CHL	3/8.82	0/0.00	3/5.56	1/16.67	0/0.00	1/5.26
	FLO	5/14.71	2/10.00	7/12.96	0/0.00	2/15.38	2/10.53
Tetracyclines	TET	30/88.24	18/90.00	48/88.89	6/100.00	10/76.92	16/84.21
Ketolides	TEL	6/17.65	0/0.00	6/11.11	4/66.67	4/30.77	8/42.11
Lincosamides	CLI	8/23.53	1/5.00	9/16.67	3/50.00	5/38.46	8/42.11

**Table 4:** Resistance spectra of 54 *C*. *jejuni* isolates to various antibiotic combinations.

MARI	No. of *C*.*coli* isolates	Antibiotic resistance patterns
0.18	5	NAL,TET
0.18	3	NAL,CIP
0.18	1	CIP,TET
0.27	27	NAL,CIP,TET
0.27	1	ERY,TEL,CLI
0.27	1	NAL,CIP,GEN
0.36	4	NAL,CIP,GEN,TET
0.36	2	NAL,CIP,FLO,TET
0.36	1	NAL,CIP,TET,CLI
0.36	1	ERY,AZT,NAL,CIP
0.45	1	NAL,CIP,FLO,TET,CHL
0.45	1	NAL,CIP,STR,FLO,TET
0.55	1	AZT,NAL,CIP,FLO,TET,CHL
0.55	1	ERY,NAL,CIP,TET,TEL,CLI
0.64	1	ERY,AZT,NAL,CIP,TET,TEL,CLI
0.82	1	ERY,AZT,GEN,STR,CHL,FLO,TET,TEL,CLI
0.91	1	ERY,AZT,NAL,CIP,GEN,STR,CHL,TET,TEL,CLI
0.91	1	ERY,AZT,NAL,CIP,STR,CHL,FLO,TET,TEL,CLI

**Table 5:** Resistance spectra of 19 *C*. *coli* isolates to various antibiotic combinations.

MARI	No. of *C*.*coli* isolates	Antibiotic resistance patterns
0.09	1	TET
0.27	1	NAL,CIP,TET
0.36	3	ERY,AZT,NAL,CIP
0.36	1	NAL,CIP,GEN,TET
0.45	1	ERY,NAL,CIP,GEN,TET
0.45	2	NAL,CIP,GEN,STR,TET
0.55	2	ERY,AZT,NAL,CIP,TET,TEL
0.55	3	ERY,NAL,CIP,TET,TEL,CLI
0.64	2	ERY,NAL,CIP,GEN,STR,FLO,TET
0.73	1	ERY,AZT,NAL,CIP,STR,TET,TEL,CLI
0.73	1	ERY,NAL,CIP,GEN,TET,TET,TEL,CLI
0.73	1	ERY,AZT,NAL,CIP,CHL,TET,TEL,CLI

## Discussion

Bacterial diarrhea poses a serious global public health challenge, with *Campylobacter* infection considered one of the primary culprits [12]. In recent years, *Campylobacter* infections have been on the rise worldwide [13]. Conventional methods for the isolation and identification of *Campylobacter* include enrichment culturing and selective isolation. The enrichment with double membrane filtration method was recognized as a more effective isolation method for *Campylobacter* and several studies of *Campylobacter* prevalence in diarrhea cases have been conducted using this method previously, with isolation ratio from 7.0%-12.1% [14–18]. Zhang et al. reported the *Campylobacter* isolation rate in diarrhea patients in Beijing was 7.81% [14]. Another study in Wenzhou (Southeast China) in patients with diarrhea showed that prevalence of Campylobacter infection was 10.5% [17]. Yan et al. revealed a *C*. *jejuni* prevalence of 4.0% in children and 5.8% in adults with diarrhea in Shenzhen (South China) [15]. The discrepancy in prevalence may be caused by regional differences or variations in sample size. Besides, picking out the suspected *Campylobacter* colonies on the selective medium was laboratory experience depending, and this might be another reason why there has been such variations in prevalence reported for diarrheal patients [16]. In this study, the double membrane filtration method was employed for the first time starting from September 2021 to detect *Campylobacter* in fecal specimens from diarrhea patients in Huzhou. The detection rate reached 11.70% among 342 diarrhea samples, surpassing the detection rates of other causative agents such as enteropathogenic *Escherichia coli* (6.49%), *Vibrio parahaemolyticus* (5.36%), and *Salmonella* (2.85%) in recent years [19,20]. *Campylobacter* has emerged as a predominant foodborne pathogen in the local region. Notably, the detection rate of *C*. *jejuni* in diarrheal patients was significantly higher than that of *C*. *coli* (χ^2^ = 20.81, P < 0.0001), consistent with previous study in other region of China [17,18]. Poultry has long been identified as the primary vehicle for sporadic *Campylobacter*iosis and the most common cause of *Campylobacter* outbreaks [17,21]. A meta-analysis by Zbrunab et al. on the global prevalence of *Campylobacter* in animal products also highlighted chickens as the primary source of *Campylobacter* transmission [22]. Similar results were also found in this study, the double membrane filtration method was applied to test 168 raw meat products (50 samples of livestork meat products and 118 samples of raw poultry products), and 38 *Campylobacter* isolates were detected, all originating from poultry meat, with chicken been the major source of infection (86.84%, 33/38).

The MLST and other genotyping approaches, including PFGE technology, shows that *Campylobacter* is not a genetically monomorphic organism, but includes highly diverse assemblies with an array of dierent phenotypes [17,23–25]. Consistent with previous reports, both PGFE type and MLST data confirmed that *Campylobacter* stains circulating in Huzhou are genetically diverse, with *C*. *jejuni* isolates being more diverse than *C*. *coli* based on MLST analysis. PFGE typing revealed 45 band patterns among 54 *C*. *jejuni* strains and 17 band patterns among 19 *C*. *coli* strains. Our findings demonstrated that the 50 *C*. *jejuni* strains from different sources were classified into 37 ST types, showing a dispersed distribution and encompassing over 12 CCs. The distribution of ST types in the 18 *C*. *coli* strains was relatively concentrated, with 83.33% (15/18) of isolates belonging to the CC-828, consistent with previous reports [26,27]. We also identified seven *C*. *jejuni* strains and one *C*. *coli* strain with novel ST types, enriching the global MLST database.

Numerous studies have reported varying major CCs of *C*. *jejuni* in different countries and regions, but CC-21, CC-45, CC-353, and CC-574 are consistently the predominant CCs among isolates in many investigations [17,24,28–30], with CC-21 considered closely associated with human infections [31] and representing 17.9% of all *C*. *jejuni* strains submitted to the PubMLST database. Our study reveals that the most prevalent CC among different sources of *C*. *jejuni* is CC-21 (22.00%, 11/50), with 9 strains from diarrhea patients and 2 strains from raw meat products. The CC-464, reported as a dominant clone in domestic settings [17], was detected in 2 strains of patients isolate and 1 strain of food isolates.

Recent phylogenetic studies using relatedness between PFGE and MLST have revealed that the two methods have effective discriminatory power in evaluating the genetic homology among *Campylobacter* strains [17,21,32]. In this study, 2 groups of *C*. *jejuni* strains (PFGE J2-ST464 and PFGE J9-ST-2328) originated from humans and chickens showed high genetic homologies by comparing PFGE and MLST results. Besides, some disagreement between PFGE and MLST was observed for certain ST, indicating a weak correlation between PFGE and MLST for certain *Campylobacter* strains. As the sequencing cost continues to decrease, next-generation sequencing (NGS) technology, which has the advantages of high throughput, high precision, and rich genetic information, may be more suitable for evaluating the genetic homology among *Campylobacter* strains from different sources.

In recent years, the widespread and sometimes inappropriate use, even misuse, of antibiotics in clinical practice and extensive long-term use of antibiotic drugs in animal husbandry have led to the emergence of antibiotic-resistant *Campylobacter* strains. According to literature reports, *Campylobacter* exhibits high resistance to quinolones and tetracycline antibiotics, with fluoroquinolone resistance rates ranging from 75% to 90% in *Campylobacter* strains from different countries [33,34]. The use of fluoroquinolones in food-producing animals has resulted in fluoroquinolone-resistant *Campylobacter* strains worldwide [35]. In this study, *C*. *jejuni* showed resistance most frequently to NAL (94.44%), followed by TET (88.89%), CIP (87.04%), CLI (16.67%), while *C*. *coli* displayed highest resistance rate to NAL/CIP (94.74%), followed by TET (84.21%). Overall, *C*. *coli* showed higher resistance rates to all antimicrobials than *C*. *jejuni* did except for CHL, FLO and TET, with MDR rate significantly higher than that of *C*. *jejuni*. Similar findings have been reported in Southeast [17] and North China [14], as well as other countries. Additionally, we observed ERY resistance in *C*. *coli* (63.16%) is much more prevalent than that in *C*. *jejuni* (63.16% vs 12.96%), in accordance with other studies [26,36–39]. The widely observed higher rate of macrolide resistance in *C*. *coli* than in *C*. *jejuni* may be associated with fitness costs impacts of certain antibiotic-resistant mutants, with the underlying mechanisms remain to be further elucidated [26,40]. In addition, over 90% of *C*. *jejuni* and *C*. *coli* clinical samples were susceptible to chloramphenicol (CHL), indicating that chloramphenicol remains effective for the treatment of *C*. *jejuni* and *C*. *coli* infection in Huzhou area.

Furthermore, it is worth mentioning that research regarding zoonotic diseases often focuses on infectious diseases animals have given to humans. However, an increasing number of reports indicate that bacteria expressing resistance to critically important antimicrobials were likely introduced along pathways involving reverse zoonosis (human-animal transmission) [41]. This included emergence of human pandemic O25:H4-ST131 CTX-M-15 extended-spectrum-beta-lactamase-producing *Escherichia coli* among companion animals [42] and community-associated methicillin-resistant Staphylococcus aureus, in dairy cow [43]. Recent reports from New Zealand demonstrated that fluoroquinolone resistance detected there among poultry was attributable to the emergence of a new clone of *C*. *jejuni* (ST6964) and it has been hypothesized that this clone was potentially introduced via exposure to other species (human or other livestock) because fluoroquinolones are not registered for use in poultry in New Zealand [44]. Therefore, the risk of antibiotic-resistant *Campylobacter* being transmitted from humans, including raw meat handlers, to poultry should not be overlooked.

## Conclusions

In conclusion, this study provides a preliminary understanding of the molecular genetic features and antibiotic resistance characteristics of *Campylobacter* spp. from raw meat products and diarrhea cases in the Huzhou area. *Campylobacter* is an important foodborne pathogen in both diarrheal patients and raw meat products in Huzhou City, exhibiting multiple antibiotic resistance and high level of genetic diversity. Two groups of *C*. *jejuni* strains originated from humans and chickens were confirmed to be clonally related by comparing PFGE and MLST results. More comprehensive study based on the genetic correlation between isolates from humans and food animals is needed to prevent and control diseases caused by them. Considering the advantages of NGS, future work is warranted to integrate NGS-based typing methods into routine foodborne pathogen surveillance to elucidate the molecular characteristics of *Campylobacter* spp. isolates.

## Supporting information

S1 Table MLST data summary of 50 *C*. *jejuni* strains and 18 *C*. *coli* strains in this study. (DOCX)

## References

[1] Vojdani A, Vojdani E. Reaction of antibodies to Campylobacter jejuni and cytolethal distending toxin B with tissues and food antigens. World journal of gastroenterology. 2019;25(9):1050–66. Epub 2019/03/14. doi: 10.3748/wjg.v25.i9.1050 ; PubMed Central PMCID: PMC6406185. 30862994 PMC6406185

[2] Langendorf C, Le Hello S, Moumouni A, Gouali M, Mamaty AA, Grais RF, et al. Enteric bacterial pathogens in children with diarrhea in Niger: diversity and antimicrobial resistance. PloS one. 2015;10(3):e0120275. Epub 2015/03/24. doi: 10.1371/journal.pone.0120275 ; PubMed Central PMCID: PMC4370739. 25799400 PMC4370739

[3] Coker AO, Isokpehi RD, Thomas BN, Amisu KO, Obi CL. Human campylobacteriosis in developing countries. Emerging infectious diseases. 2002;8(3):237–44. doi: 10.3201/eid0803.010233 ; PubMed Central PMCID: PMC2732465. 11927019 PMC2732465

[4] Ruiz-Palacios GM. The health burden of Campylobacter infection and the impact of antimicrobial resistance: playing chicken. Clinical infectious diseases: an official publication of the Infectious Diseases Society of America. 2007;44(5):701–3. Epub 2007/02/06. doi: 10.1086/509936 . 17278063

[5] Mead GC, Hudson WR, Hinton MH. Effect of changes in processing to improve hygiene control on contamination of poultry carcasses with campylobacter. Epidemiology and infection. 1995;115(3):495–500. Epub 1995/12/01. doi: 10.1017/s0950268800058659 ; PubMed Central PMCID: PMC2271579. 8557081 PMC2271579

[6] Lin J. Novel approaches for Campylobacter control in poultry. Foodborne pathogens and disease. 2009;6(7):755–65. doi: 10.1089/fpd.2008.0247 ; PubMed Central PMCID: PMC3145176. 19425824 PMC3145176

[7] Wilson DJ, Gabriel E, Leatherbarrow AJ, Cheesbrough J, Gee S, Bolton E, et al. Tracing the source of campylobacteriosis. PLoS genetics. 2008;4(9):e1000203. Epub 2008/09/27. doi: 10.1371/journal.pgen.1000203 ; PubMed Central PMCID: PMC2538567. 18818764 PMC2538567

[8] Paudyal N, Pan H, Liao X, Zhang X, Li X, Fang W, et al. A Meta-Analysis of Major Foodborne Pathogens in Chinese Food Commodities Between 2006 and 2016. Foodborne pathogens and disease. 2018;15(4):187–97. doi: 10.1089/fpd.2017.2417 . 29652195

[9] Kuhn KG, Falkenhorst G, Emborg HD, Ceper T, Torpdahl M, Krogfelt KA, et al. Epidemiological and serological investigation of a waterborne Campylobacter jejuni outbreak in a Danish town. Epidemiology and infection. 2017;145(4):701–9. Epub 20161201. doi: 10.1017/S0950268816002788 ; PubMed Central PMCID: PMC9507752. 27903324 PMC9507752

[10] Burakoff A, Brown K, Knutsen J, Hopewell C, Rowe S, Bennett C, et al. Outbreak of Fluoroquinolone-Resistant Campylobacter jejuni Infections Associated with Raw Milk Consumption from a Herdshare Dairy—Colorado, 2016. MMWR Morb Mortal Wkly Rep. 2018;67(5):146–8. Epub 20180209. doi: 10.15585/mmwr.mm6705a2 ; PubMed Central PMCID: PMC5812469. 29420460 PMC5812469

[11] Chen H, Dai Y, Chen J, Zhang Y, Zhan L, Mei L, et al. Epidemiological and Whole Genomic Sequencing Analysis of a Campylobacter jejuni Outbreak in Zhejiang Province, China, May 2019. Foodborne pathogens and disease. 2020;17(12):775–81. Epub 20200707. doi: 10.1089/fpd.2020.2794 . 32639172

[12] Pavlova MR, Dobreva EG, Ivanova KI, Asseva GD, Ivanov IN, Petrov PK, et al. Multiplex PCR Assay for Identifi cation and Differentiation of Campylobacter jejuni and Campylobacter coli Isolates. Folia medica. 2016;58(2):95–100. Epub 2016/08/24. doi: 10.1515/folmed-2016-0016 . 27552785

[13] Kaakoush NO, Castaño-Rodríguez N, Mitchell HM, Man SM. Global Epidemiology of Campylobacter Infection. Clinical microbiology reviews. 2015;28(3):687–720. Epub 2015/06/13. doi: 10.1128/CMR.00006-15 ; PubMed Central PMCID: PMC4462680. 26062576 PMC4462680

[14] Zhang P, Zhang X, Liu Y, Jiang J, Shen Z, Chen Q, et al. Multilocus Sequence Types and Antimicrobial Resistance of Campylobacter jejuni and C. coli Isolates of Human Patients From Beijing, China, 2017–2018. Frontiers in microbiology. 2020;11:554784. Epub 2020/11/17. doi: 10.3389/fmicb.2020.554784 ; PubMed Central PMCID: PMC7604515. 33193135 PMC7604515

[15] Ju CY, Zhang MJ, Ma YP, Lu JR, Yu MH, Chen H, et al. Genetic and Antibiotic Resistance Characteristics of Campylobacter jejuni Isolated from Diarrheal Patients, Poultry and Cattle in Shenzhen. Biomed Environ Sci. 2018;31(8):579–85. doi: 10.3967/bes2018.079 ; PubMed Central PMCID: PMC6766747. 30231962 PMC6766747

[16] Liang H, Wen Z, Li Y, Duan Y, Gu Y, Zhang M. Comparison of the Filtration Culture and Multiple Real-Time PCR Examination for Campylobacter spp. From Stool Specimens in Diarrheal Patients. Frontiers in microbiology. 2018;9:2995. Epub 20181205. doi: 10.3389/fmicb.2018.02995 ; PubMed Central PMCID: PMC6290255. 30568645 PMC6290255

[17] Zhang L, Li Y, Shao Y, Hu Y, Lou H, Chen X, et al. Molecular Characterization and Antibiotic Resistant Profiles of Campylobacter Species Isolated From Poultry and Diarrheal Patients in Southeastern China 2017–2019. Frontiers in microbiology. 2020;11:1244. Epub 2020/07/14. doi: 10.3389/fmicb.2020.01244 ; PubMed Central PMCID: PMC7324532. 32655522 PMC7324532

[18] Li Y, Zhang S, He M, Zhang Y, Fu Y, Liang H, et al. Prevalence and Molecular Characterization of Campylobacter spp. Isolated from Patients with Diarrhea in Shunyi, Beijing. Frontiers in microbiology. 2018;9:52. Epub 2018/02/13. doi: 10.3389/fmicb.2018.00052 ; PubMed Central PMCID: PMC5790792. 29434579 PMC5790792

[19] Zhang P, Wu X, Yuan R, Yan W, Xu D, Ji L, et al. Emergence and predominance of a new serotype of Vibrio parahaemolyticus in Huzhou, China. International journal of infectious diseases: IJID: official publication of the International Society for Infectious Diseases. 2022;122:93–8. Epub 2022/05/15. doi: 10.1016/j.ijid.2022.05.023 . 35568367

[20] Xiaofang-W, Deshun-X, Lei-J, Yunfeng-Z, Liping-C.—Surveillance results of foodborne diseases in Huzhou, Zhejiang, 2018–2020.—Disease Surveillance. 2021;- 36(- 9):- 958. doi: 10.3784/jbjc.202105150270

[21] Oh JY, Kwon YK, Wei B, Jang HK, Lim SK, Kim CH, et al. Epidemiological relationships of Campylobacter jejuni strains isolated from humans and chickens in South Korea. Journal of microbiology (Seoul, Korea). 2017;55(1):13–20. Epub 2016/12/31. doi: 10.1007/s12275-017-6308-8 . 28035601

[22] Zbrun MV, Rossler E, Romero-Scharpen A, Soto LP, Berisvil A, Zimmermann JA, et al. Worldwide meta-analysis of the prevalence of Campylobacter in animal food products. Research in veterinary science. 2020;132:69–77. Epub 2020/06/11. doi: 10.1016/j.rvsc.2020.05.017 . 32521281

[23] Duarte A, Seliwiorstow T, Miller WG, De Zutter L, Uyttendaele M, Dierick K, et al. Discriminative power of Campylobacter phenotypic and genotypic typing methods. Journal of microbiological methods. 2016;125:33–9. Epub 2016/03/22. doi: 10.1016/j.mimet.2016.03.004 . 26996762

[24] Zhang D, Zhang X, Lyu B, Tian Y, Huang Y, Lin C, et al. Genomic Analysis and Antimicrobial Resistance of Campylobacter jejuni Isolated from Diarrheal Patients—Beijing Municipality, China, 2019–2021. China CDC weekly. 2023;5(19):424–33. Epub 2023/06/05. doi: 10.46234/ccdcw2023.080 ; PubMed Central PMCID: PMC10235816. 37275268 PMC10235816

[25] Yu H, Elbediwi M, Zhou X, Shuai H, Lou X, Wang H, et al. Epidemiological and Genomic Characterization of Campylobacter jejuni Isolates from a Foodborne Outbreak at Hangzhou, China. International journal of molecular sciences. 2020;21(8). Epub 2020/04/30. doi: 10.3390/ijms21083001 ; PubMed Central PMCID: PMC7215453. 32344510 PMC7215453

[26] Gao F, Tu L, Chen M, Chen H, Zhang X, Zhuang Y, et al. Erythromycin resistance of clinical Campylobacter jejuni and Campylobacter coli in Shanghai, China. Frontiers in microbiology. 2023;14:1145581. Epub 2023/06/01. doi: 10.3389/fmicb.2023.1145581 ; PubMed Central PMCID: PMC10229067. 37260688 PMC10229067

[27] Zhang M, Liu X, Xu X, Gu Y, Tao X, Yang X, et al. Molecular subtyping and antimicrobial susceptibilities of Campylobacter coli isolates from diarrheal patients and food-producing animals in China. Foodborne pathogens and disease. 2014;11(8):610–9. Epub 2014/05/23. doi: 10.1089/fpd.2013.1721 . 24844559

[28] Niederer L, Kuhnert P, Egger R, Büttner S, Hächler H, Korczak BM. Genotypes and antibiotic resistances of Campylobacter jejuni and Campylobacter coli isolates from domestic and travel-associated human cases. Applied and environmental microbiology. 2012;78(1):288–91. Epub 2011/10/25. doi: 10.1128/AEM.06194-11 ; PubMed Central PMCID: PMC3255622. 22020515 PMC3255622

[29] Colles FM, Maiden MCJ. Campylobacter sequence typing databases: applications and future prospects. Microbiology (Reading, England). 2012;158(Pt 11):2695–709. Epub 2012/09/19. doi: 10.1099/mic.0.062000-0 . 22986295

[30] Prachantasena S, Charununtakorn P, Muangnoicharoen S, Hankla L, Techawal N, Chaveerach P, et al. Distribution and Genetic Profiles of Campylobacter in Commercial Broiler Production from Breeder to Slaughter in Thailand. PloS one. 2016;11(2):e0149585. Epub 2016/02/18. doi: 10.1371/journal.pone.0149585 ; PubMed Central PMCID: PMC4757449. 26886590 PMC4757449

[31] Lévesque S, Frost E, Arbeit RD, Michaud S. Multilocus sequence typing of Campylobacter jejuni isolates from humans, chickens, raw milk, and environmental water in Quebec, Canada. Journal of clinical microbiology. 2008;46(10):3404–11. Epub 2008/08/15. doi: 10.1128/JCM.00042-08 ; PubMed Central PMCID: PMC2566118. 18701662 PMC2566118

[32] Behringer M, Miller WG, Oyarzabal OA. Typing of Campylobacter jejuni and Campylobacter coli isolated from live broilers and retail broiler meat by flaA-RFLP, MLST, PFGE and REP-PCR. Journal of microbiological methods. 2011;84(2):194–201. Epub 2010/12/07. doi: 10.1016/j.mimet.2010.11.016 . 21130125

[33] Schiaffino F, Colston JM, Paredes-Olortegui M, François R, Pisanic N, Burga R, et al. Antibiotic Resistance of Campylobacter Species in a Pediatric Cohort Study. Antimicrobial agents and chemotherapy. 2019;63(2). Epub 2018/11/14. doi: 10.1128/aac.01911-18 ; PubMed Central PMCID: PMC6355604. 30420482 PMC6355604

[34] Signorini ML, Rossler E, Díaz David DC, Olivero CR, Romero-Scharpen A, Soto LP, et al. Antimicrobial Resistance of Thermotolerant Campylobacter Species Isolated from Humans, Food-Producing Animals, and Products of Animal Origin: A Worldwide Meta-Analysis. Microbial drug resistance (Larchmont, NY). 2018;24(8):1174–90. Epub 2018/05/01. doi: 10.1089/mdr.2017.0310 . 29708832

[35] Hannula M, Hänninen ML. Effects of low-level ciprofloxacin challenge in the in vitro development of ciprofloxacin resistance in Campylobacter jejuni. Microbial drug resistance (Larchmont, NY). 2008;14(3):197–201. Epub 2008/08/21. doi: 10.1089/mdr.2008.0833 . 18713066

[36] Florez-Cuadrado D, Ugarte-Ruiz M, Meric G, Quesada A, Porrero MC, Pascoe B, et al. Genome Comparison of Erythromycin Resistant Campylobacter from Turkeys Identifies Hosts and Pathways for Horizontal Spread of erm(B) Genes. Frontiers in microbiology. 2017;8:2240. Epub 2017/12/01. doi: 10.3389/fmicb.2017.02240 ; PubMed Central PMCID: PMC5695097. 29187841 PMC5695097

[37] The European Union Summary Report on Antimicrobial Resistance in zoonotic and indicator bacteria from humans, animals and food in 2019–2020. EFSA journal European Food Safety Authority. 2022;20(3):e07209. Epub 2022/04/07. doi: 10.2903/j.efsa.2022.7209 ; PubMed Central PMCID: PMC8961508. 35382452 PMC8961508

[38] Liao YS, Chen BH, Teng RH, Wang YW, Chang JH, Liang SY, et al. Antimicrobial Resistance in Campylobacter coli and Campylobacter jejuni from Human Campylobacteriosis in Taiwan, 2016 to 2019. Antimicrobial agents and chemotherapy. 2022;66(1):e0173621. Epub 2021/11/09. doi: 10.1128/AAC.01736-21 ; PubMed Central PMCID: PMC8765299. 34748382 PMC8765299

[39] Quino W, Caro-Castro J, Hurtado V, Flores-León D, Gonzalez-Escalona N, Gavilan RG. Genomic Analysis and Antimicrobial Resistance of Campylobacter jejuni and Campylobacter coli in Peru. Frontiers in microbiology. 2021;12:802404. Epub 2022/01/29. doi: 10.3389/fmicb.2021.802404 ; PubMed Central PMCID: PMC8787162. 35087501 PMC8787162

[40] Bolinger H, Kathariou S. The Current State of Macrolide Resistance in Campylobacter spp.: Trends and Impacts of Resistance Mechanisms. Applied and environmental microbiology. 2017;83(12). Epub 2017/04/16. doi: 10.1128/AEM.00416-17 ; PubMed Central PMCID: PMC5452823. 28411226 PMC5452823

[41] Messenger AM, Barnes AN, Gray GC. Reverse zoonotic disease transmission (zooanthroponosis): a systematic review of seldom-documented human biological threats to animals. PloS one. 2014;9(2):e89055. Epub 20140228. doi: 10.1371/journal.pone.0089055 ; PubMed Central PMCID: PMC3938448. 24586500 PMC3938448

[42] Ewers C, Grobbel M, Stamm I, Kopp PA, Diehl I, Semmler T, et al. Emergence of human pandemic O25:H4-ST131 CTX-M-15 extended-spectrum-beta-lactamase-producing Escherichia coli among companion animals. J Antimicrob Chemother. 2010;65(4):651–60. Epub 20100129. doi: 10.1093/jac/dkq004 . 20118165

[43] Abraham S, Jagoe S, Pang S, Coombs GW, O’Dea M, Kelly J, et al. Reverse zoonotic transmission of community-associated MRSA ST1-IV to a dairy cow. Int J Antimicrob Agents. 2017;50(1):125–6. Epub 20170511. doi: 10.1016/j.ijantimicag.2017.05.001 . 28502696

[44] Abraham S, Sahibzada S, Hewson K, Laird T, Abraham R, Pavic A, et al. Emergence of Fluoroquinolone-Resistant Campylobacter jejuni and Campylobacter coli among Australian Chickens in the Absence of Fluoroquinolone Use. Applied and environmental microbiology. 2020;86(8). Epub 20200401. doi: 10.1128/AEM.02765-19 ; PubMed Central PMCID: PMC7117913. 32033955 PMC7117913

