# Supplementary material for: Molecular typing and antimicrobial susceptibility profiles of Campylobacter jejuni and Campylobacter coli Isolates from Patients and raw meat in Huzhou, China, 2021–2022
Source: PLoS One. 2024 Dec 11;19(12):e0311769. doi: 10.1371/journal.pone.0311769 (PMC11633965; doi:10.1371/journal.pone.0311769)
Supplement: S1 Table — (DOCX) [file pone.0311769.s001.docx]

| Strain | Specimen origin | aspA | glnA | gltA | glyA | pgm | tkt | uncA | ST | CC | Year | Species type |
| --- | --- | --- | --- | --- | --- | --- | --- | --- | --- | --- | --- | --- |
| HuZ22016 | Patient | 9 | 17 | 5 | 10 | 350 | 3 | 3 | 2274 | none | 2022 | *Campylobacter jejuni* |
| HuZ22126 | Patient | 1 | 2 | 2 | 10 | 11 | 5 | 6 | 12391* | none | 2022 | *Campylobacter jejuni* |
| HuZ22127 | Patient | 1 | 2 | 2 | 10 | 11 | 5 | 6 | 12391* | none | 2022 | *Campylobacter jejuni* |
| HuZ22129 | Patient | 9 | 1 | 12 | 3 | 2 | 1 | 5 | 298 | 21 | 2022 | *Campylobacter jejuni* |
| HuZ22130 | Patient | 60 | 69 | 52 | 10 | 90 | 3 | 6 | 4268 | none | 2022 | *Campylobacter jejuni* |
| HuZ22140 | Patient | 24 | 2 | 2 | 2 | 10 | 3 | 1 | [464](https://pubmlst.org/bigsdb?page=profileInfo&db=pubmlst_campylobacter_seqdef&scheme_id=1&profile_id=464) | 464 | 2022 | *Campylobacter jejuni* |
| HuZ22141 | Patient | 7 | 2 | 5 | 2 | 1093 | 3 | 6 | 10727 | 353 | 2022 | *Campylobacter jejuni* |
| HuZ22145 | Patient | 2 | 1 | 52 | 3 | 1196 | 100 | 5 | 11822* | 21 | 2022 | *Campylobacter jejuni* |
| HuZ22146 | Patient | 7 | 2 | 5 | 10 | 10 | 37 | 1 | 1035 | none | 2022 | *Campylobacter jejuni* |
| HuZ22226 | Patient | 166 | 1 | 1 | 10 | 22 | 3 | 1 | 11855 | none | 2022 | *Campylobacter jejuni* |
| HuZ22272 | Patient | 9 | 1 | 12 | 3 | 2 | 1 | 5 | 298 | 21 | 2022 | *Campylobacter jejuni* |
| HuZ22273 | Patient | 9 | 1 | 12 | 3 | 2 | 1 | 5 | 298 | 21 | 2022 | *Campylobacter jejuni* |
| HuZ22274 | Patient | 7 | 17 | 358 | 2 | 22 | 3 | 1 | 7433 | 353 | 2022 | *Campylobacter jejuni* |
| HuZ22278 | Patient | 8 | 10 | 2 | 2 | 11 | 12 | 6 | 354 | 354 | 2022 | *Campylobacter jejuni* |
| HuZ22279 | Patient | 7 | 71 | 5 | 10 | 188 | 67 | 26 | 12392* | 21 | 2022 | *Campylobacter jejuni* |
| HuZ22280 | Patient | 7 | 71 | 5 | 10 | 188 | 67 | 26 | 12392* | 21 | 2022 | *Campylobacter jejuni* |
| HuZ22356 | Patient | 9 | 1 | 12 | 3 | 2 | 1 | 5 | 298 | 21 | 2022 | *Campylobacter jejuni* |
| HuZ22636 | Patient | 8 | 2 | 2 | 212 | 153 | 253 | 147 | 2328 | none | 2022 | *Campylobacter jejuni* |
| HuZ22637 | Patient | 9 | 2 | 4 | 62 | 4 | 133 | 6 | 990 | 257 | 2022 | *Campylobacter jejuni* |
| HuZ22728 | Patient | 7 | 4 | 2 | 68 | 11 | 1 | 6 | 11711 | none | 2022 | *Campylobacter jejuni* |
| HuZ22729 | Patient | 3 | 1 | 5 | 17 | 11 | 11 | 6 | 49 | 49 | 2022 | *Campylobacter jejuni* |
| HuZ22747 | Patient | 37 | 364 | 4 | 734 | 127 | 24 | 5 | 12334 | none | 2022 | *Campylobacter jejuni* |
| HuZ22813 | Patient | 9 | 17 | 5 | 10 | 11 | 3 | 3 | 2031 | 574 | 2022 | *Campylobacter jejuni* |
| HuZ22814 | Patient | 9 | 2 | 5 | 2 | 11 | 3 | 6 | 2842 | 353 | 2022 | *Campylobacter jejuni* |
| HuZ22834 | Patient | 2 | 1 | 52 | 3 | 23 | 100 | 5 | 6500 | 21 | 2022 | *Campylobacter jejuni* |
| HuZ22900 | Patient | 4 | 7 | 10 | 4 | 89 | 51 | 1 | 12371* | 45 | 2022 | *Campylobacter jejuni* |
| HuZ21483 | Patient | 24 | 2 | 2 | 2 | 10 | 3 | 1 | 464 | 464 | 2021 | *Campylobacter jejuni* |
| HuZ21739 | Patient | 9 | 53 | 2 | 10 | 11 | 3 | 3 | 305 | 574 | 2021 | *Campylobacter jejuni* |
| HuZ21740 | Patient | 24 | 2 | 2 | 72 | 22 | 406 | 6 | 4327 | none | 2021 | *Campylobacter jejuni* |
| HuZ21762 | Patient | 593 | 1 | 5 | 17 | 11 | 11 | 6 | 11775* | 49 | 2021 | *Campylobacter jejuni* |
| HuZ21763 | Patient | 2 | 1 | 5 | 10 | 608 | 1 | 5 | 6175 | 21 | 2021 | *Campylobacter jejuni* |
| HuZ21764 | Patient | 8 | 2 | 27 | 751 | 22 | 3 | 1 | 9621 | 607 | 2021 | *Campylobacter jejuni* |
| HuZ21768 | Patient | 4 | 7 | 10 | 4 | 42 | 51 | 1 | 583 | 45 | 2021 | *Campylobacter jejuni* |
| HuZ21770 | Patient | 55 | 21 | 2 | 71 | 11 | 37 | 3 | 2133 | none | 2021 | *Campylobacter jejuni* |
| HuZ22128 | Patient | 32 | 42 | 30 | 79 | 104 | 47 | 17 | 11932 | none | 2022 | *Campylobacter coli* |
| HuZ22131 | Patient | 33 | 38 | 30 | 82 | 113 | 43 | 17 | 1563 | 828 | 2022 | *Campylobacter coli* |
| HuZ22523 | Patient | 82 | 38 | 30 | 82 | 104 | 43 | 17 | 12390* | 828 | 2022 | *Campylobacter coli* |

| Strain | Specimen origin | aspA | glnA | gltA | glyA | pgm | tkt | uncA | ST | CC | Year | Species type |
| --- | --- | --- | --- | --- | --- | --- | --- | --- | --- | --- | --- | --- |
| HuZ22730 | Patient | 33 | 42 | 30 | 82 | 104 | 47 | 17 | 9743 | 828 | 2022 | *Campylobacter coli* |
| HuZ21738 | Patient | 33 | 39 | 30 | 82 | 113 | 47 | 139 | 5511 | 828 | 2021 | *Campylobacter coli* |
| HuZ21769 | Patient | 33 | 39 | 30 | 82 | 113 | 47 | 17 | 825 | 828 | 2021 | *Campylobacter coli* |
| HuZ22073 | Fresh Chicken | 8 | 10 | 2 | 2 | 10 | 12 | 6 | 2988 | 354 | 2022 | *Campylobacter jejuni* |
| HuZ22074 | Fresh Chicken | 7 | 30 | 2 | 2 | 89 | 59 | 6 | 1213 | 460 | 2022 | *Campylobacter jejuni* |
| HuZ22753 | Fresh Chicken | 2 | 609 | 12 | 3 | 2 | 3 | 5 | 8261 | 21 | 2022 | *Campylobacter jejuni* |
| HuZ22755 | Fresh Duck | 2 | 61 | 4 | 64 | 1 | 7 | 23 | 6040 | 1034 | 2022 | *Campylobacter jejuni* |
| HuZ22756 | Fresh Chicken | 8 | 2 | 2 | 212 | 153 | 253 | 147 | 2328 | none | 2022 | *Campylobacter jejuni* |
| HuZ22768 | Frozen Chicken CChickenhicken | 166 | 2 | 1 | 10 | 127 | 59 | 1 | 6606 | none | 2022 | *Campylobacter jejuni* |
| HuZ22770 | Frozen Chicken | 8 | 2 | 27 | 751 | 22 | 3 | 1 | 9621 | 607 | 2022 | *Campylobacter jejuni* |
| HuZ22772 | Fresh Chicken | 7 | 17 | 2 | 10 | 11 | 3 | 12 | 8880 | 574 | 2022 | *Campylobacter jejuni* |
| HuZ22282 | Fresh Chicken | 14 | 21 | 2 | 10 | 86 | 3 | 6 | 161 | 52 | 2022 | *Campylobacter jejuni* |
| HuZ22284 | Fresh Duck | 2 | 1 | 52 | 3 | 23 | 100 | 5 | 6500 | 21 | 2022 | *Campylobacter jejuni* |
| HuZ22731 | Fresh Chicken | 8 | 2 | 2 | 212 | 153 | 253 | 147 | 2328 | none | 2022 | *Campylobacter jejuni* |
| HuZ22732 | Fresh Chicken | 9 | 22 | 173 | 146 | 11 | 3 | 6 | 6607 | none | 2022 | *Campylobacter jejuni* |
| HuZ22734 | Fresh Chicken | 8 | 2 | 2 | 212 | 153 | 253 | 147 | 2328 | none | 2022 | *Campylobacter jejuni* |
| HuZ22735 | Fresh Chicken | 14 | 21 | 2 | 10 | 86 | 3 | 6 | 161 | 52 | 2022 | *Campylobacter jejuni* |
| HuZ22742 | Fresh Chicken | 14 | 2 | 6 | 15 | 914 | 3 | 6 | 9138 | none | 2022 | *Campylobacter jejuni* |
| HuZ22744 | Fresh Chicken | 24 | 2 | 2 | 2 | 10 | 3 | 1 | 464 | 464 | 2022 | *Campylobacter jejuni* |
| HuZ22737 | Fresh Duck | 33 | 39 | 30 | 82 | 104 | 44 | 33 | 1145 | none | 2022 | *Campylobacter coli* |
| HuZ22740 | Fresh Chicken | 33 | 39 | 30 | 82 | 113 | 47 | 33 | 825 | 828 | 2022 | *Campylobacter coli* |
| HuZ22741 | Fresh Chicken | 33 | 39 | 30 | 82 | 113 | 43 | 33 | 829 | 828 | 2022 | *Campylobacter coli* |
| HuZ22745 | Fresh Chicken | 33 | 39 | 30 | 82 | 104 | 43 | 33 | 828 | 828 | 2022 | *Campylobacter coli* |
| HuZ22760 | Fresh Chicken | 33 | 39 | 30 | 82 | 113 | 47 | 33 | 825 | 828 | 2022 | *Campylobacter coli* |
| HuZ22761 | Fresh Chicken | 33 | 39 | 30 | 82 | 113 | 44 | 33 | 825 | 828 | 2022 | *Campylobacter coli* |
| HuZ22769 | Frozen Chicken | 33 | 39 | 30 | 82 | 113 | 47 | 33 | 825 | 828 | 2022 | *Campylobacter coli* |
| HuZ22757 | Fresh Chicken | 33 | 39 | 30 | 79 | 104 | 43 | 33 | 902 | 828 | 2022 | *Campylobacter coli* |
| HuZ22758 | Fresh Chicken | 33 | 39 | 30 | 79 | 104 | 43 | 33 | 902 | 828 | 2022 | *Campylobacter coli* |
| HuZ22733 | Fresh Chicken | 33 | 39 | 30 | 82 | 113 | 43 | 33 | 829 | 828 | 2022 | *Campylobacter coli* |
| HuZ22739 | Fresh Chicken | 33 | 39 | 30 | 82 | 104 | 43 | 33 | 828 | 828 | 2022 | *Campylobacter coli* |
| HuZ22743 | Fresh Chicken | 32 | 39 | 30 | 79 | 104 | 47 | 32 | 12337 | none | 2022 | *Campylobacter coli* |
